# Effects of S-Allylcysteine-Rich Garlic Extract and Dietary Inorganic Nitrate Formula on Blood Pressure and Salivary Nitric Oxide: An Open-Label Clinical Trial Among Hypertensive Subjects

**DOI:** 10.7759/cureus.45369

**Published:** 2023-09-16

**Authors:** Mark Houston, Chen Chen, Christopher R D'Adamo, Adonia E Papathanassiu, Shawn J Green

**Affiliations:** 1 Cardiology, Hypertension Institute at Saint Thomas West Hospital, Nashville, USA; 2 Nutrition, Calroy Health Sciences, Greensboro, USA; 3 Family and Community Medicine, University of Maryland Medical Center, Baltimore, USA; 4 Nutrition, MyFitStrip, Rockville, USA; 5 Cardiology, Lundquist Institute at Harbor-UCLA (University of California, Los Angeles) Medical Center, Torrance, USA

**Keywords:** s-allylcysteine, garlic, oral microbiome, dietary supplementation, alternative medicine, saliva testing, hypertension, hydrogen sulfide, dietary nitrate, nitric oxide

## Abstract

Introduction: The conversion of dietary inorganic nitrate (NO3-) to nitric oxide (NO) is a non-canonical pathway that plays an important role in NO biology, especially under pathological conditions. Inorganic NO3- supplementation is a proven method for controlling mild hypertension. Recent reports have suggested that another gaseous transmitter, hydrogen sulfide (H2S), influences NO biosynthesis and metabolism. Here, data are presented from an open-label clinical trial examining the effect of an encapsulated formulation (Vascanox® HP) that combines dietary sources of inorganic NO3- and S-allylcysteine (SAC), a source of H2S from garlic, on NO bioavailability and blood pressure in subjects experiencing elevated blood pressure or mild hypertension.

Methods: An open-label clinical trial was conducted among patients with hypertension. Participants took Vascanox® for four weeks. Blood pressure was measured at baseline, two weeks, and four weeks. Salivary nitrite (NO2-), a surrogate of NO bioavailability, and NO3- were assessed prior to and two, six, and 24 hours after dosing on the first day of the study and prior to and two hours after dosing at subsequent study visits using saliva NO test strips. Changes in study outcomes over time were evaluated via analysis of variance (ANOVA) and paired t-tests.

Results: Twelve participants completed the clinical trial. Vascanox® HP decreased systolic blood pressure by ~11 mmHg (p < 0.001) at two weeks and persisted beyond four weeks with daily supplementation. It also decreased the diastolic blood pressure of hypertensive subjects but not normotensive ones. The magnitude of the decrease was 11 mmHg (p < 0.01) at four weeks of study. Measurements of salivary concentrations of NO2- revealed high peak levels (743 uM) at two hours post-administration and a slow decay to elevated levels (348 uM) at 24 hours. NO2- salivary concentrations, a surrogate biomarker of NO bioavailability, remained above baseline for the duration of the study.

Conclusions: Vascanox® HP was shown to be a safe, effective, quick-acting, and long-lasting dietary supplement for controlling mild hypertension.

## Introduction

Elevated blood pressure (BP) in the absence of an underlying pathology (or primary hypertension) is the most common medical diagnosis in the US and a major risk factor for cardiovascular disease [1]. According to recent data, one in two adults in the US suffers from hypertension [2]; the majority (>90%) of these cases are of unknown etiology, although genetic, environmental, and behavioral characteristics are thought to be contributing factors [3]. Despite the wide availability of pharmaceutical interventions, hypertension is linked to >670,000 deaths annually and poor BP control remains the most significant cause of cardiovascular mortality [4].

Primary hypertension is associated with a decline in vasodilation due to a dysfunctional endothelium that synthesizes decreased levels of nitric oxide (NO) [5]. NO mediates relaxation of smooth muscle through a mechanism that involves its binding to a heme moiety and activation of soluble guanylyl cyclase (sGC) and increases cyclic guanosine 3',5'-monophosphate (cGMP), resulting in the activation of the cGMP-dependent protein kinase G (PKG) and the ensuing release of calcium from intracellular stores in smooth muscle cells, which then leads to vasodilation [6].

NO is produced endogenously as a by-product of the oxidation of L-arginine to L-citrulline, a reaction that is catalyzed by the endothelial nitric oxide synthase (eNOS). Endogenous NO has a short half-life (t1/2 = 1-2 ms) and is rapidly oxidized to form nitrite (NO2-) with a half-life of 20-45 minutes and then nitrate (NO3-) with a half-life of five to eight hours [7,8]. The conversion of NO to NO2- and NO3- is reversible and enzymatic and non-enzymatic systems exist that reduce nitrite and nitrate to NO under certain environmental conditions [9,10]. Consequently, NO is seen as a gaseous transmitter that acts near the site of its synthesis and requires a constant source of production for systemic vasodilation, whereas nitrate with its long half-life is seen as a circulating reservoir of NO.

NO3-, formed endogenously by oxidation of NO, is also supplemented by the ingestion of nitrate-rich foods (for example, beets and green, leafy vegetables), which serve as an exogenous source of NO [8-11]. The canonical eNOS-dependent pathway and the NO3-to-NO conversion system appear to work in tandem to maintain NO homeostasis [10-13]. It should be noted here that NO3- conversion to NO requires the presence of a unique enterosalivary cycle that ensures the efficient recycling of NO3- to bioactive NO and other reactive nitrogen species (RNS). In that cycle, dietary NO3- is rapidly absorbed by the GI tract, enters the circulation, and is either excreted through the kidneys or uptaken by the salivary gland. It is estimated that ~70% of circulating nitrate is excreted whereas ~25% is found in the saliva [10-13]. As a result, the levels of nitrate in the saliva are 10-20-fold higher than those in the blood. Salivary NO3- is reduced to NO2- by the action of nitrate reductases found in a community of commensal bacteria that are particularly enriched with *Rothia mucilaginosa*, *Haemophilus parainfluenzae*, *Neisseria flavescens*, and *Neisseria subflava* [11]. Swallowed saliva then delivers NO2- into the stomach, where it is reduced to NO by the acidic environment, in the presence of vitamin C, and enters the circulation [8-10]. Several mechanisms exist to locally convert nitrite to NO such as hypoxic acidic conditions and possibly by the multifunctional enzyme xanthine oxidoreductase (XOR) [10].

Recent literature suggests that another gaseous transmitter, hydrogen sulfide (H2S), plays an essential role in maintaining NO homeostasis and proper vasodilation [6,14]. It has been known for a long time that the human body produces H2S, presumably as a metabolic waste, enzymatically in the transsulfuration pathway. Specifically, H2S is synthesized primarily from L-cysteine or homocysteine through the action of one of three enzymes: cytosolic enzymes cystathionine-synthase (CBS) and cystathionine-lyase (CGL) and the mitochondrial enzyme 3-mercaptopyruvate sulfurtransferase (MST) [6]. Non-enzymatic pathways to generate H2S from various sulfur-containing compounds also exist.

Dietary sources of H2S include garlic as a rich source [15,16]. S-allylcysteine sulfoxide and its derivative allicin, found in garlic, as well as the more stable S-allylcysteine (SAC), found in fermented black garlic, act as H2S donors. Conversion of these polysulfides to H2S is thought to take place primarily in red blood cells (RBCs) and to involve cytosolic glutathione [15]. Released H2S mediates vasorelaxation through multiple NO-dependent mechanisms. For example, it is well-understood that H2S enhances eNOS function through stimulation of the phosphoinositide 3-kinase/protein kinase B (PI3K/AKT) pathway in endothelial cells and extends the bioavailability of NO-derived cGMP by inhibiting phosphodiesterase 5A (PDE5A) [6]. It has been demonstrated that H2S shifts XOR activity to nitrite reductase to form NO [17]. Additionally, H2S has been shown to chemically interact with oxidized forms of NO to produce intermediates that serve as their own unique reservoirs for NO (e.g., S-nitrosothiols) or exert their own cardioprotective effects (e.g., nitroxyl) [18,19]. It has been reported that H2S can further interact with S-nitrosothiols to form compounds such as nitrosopersulfide (SSNO-), which are stable at physiological pH and generate NO and polysulfides upon decomposition [19]. The recognition that H2S plays an important role in vasodilation has led to the proposition that primary hypertension is the result of the simultaneous decline in the bioavailability of both NO and H2S [5].

Here, we present data from a small, open-label study that aims to begin to evaluate the durability of an encapsulated H2S-NO donor supplement on the systolic and diastolic BP of human subjects with elevated BP. The encapsulated formulation combines natural reductants and polyphenols with dietary sources of NO3- and H2S and may represent an increment alternative to current encapsulated NO3- supplementation by simultaneously restoring NO and H2S bioavailability in the vasculature.

## Materials and methods

Study population

The study was conducted in accordance with the Declaration of Helsinki and approved by the Salus Institutional Review Board (Salus IRB) affiliated with the Association for the Accreditation of Human Research Protection Programs (AAHRPP) for studies involving humans. The Salus IRB approval # is HTI-005. Informed consent was obtained from all subjects involved in the study at the Hypertensive Institute, Nashville, TN under the supervision of Dr. Mark Houston, MD. Written informed consent has been obtained from the patient(s) to publish this paper.

This study is registered with ClinicalTrials.gov (ID: NCT05928676, Unique Protocol ID: 022-CalroyHS), under the following title: Open Label Clinical Trial of Vascanox® HP on Nitric Oxide and Blood Pressure (Official Title: Effects of S-Allylcysteine-Rich Garlic Extract and Dietary Inorganic Nitrate Formula (Vascanox®) on Blood Pressure and Nitric Oxide Levels: An Open-Label Clinical Trial Among Hypertensive Adults).

The eligibility criteria for participation were: (a) elevated BP at baseline (>120-140/85-90 mmHg), (b) the ability to provide informed consent, (c) the absence of any significant cardiac or other medical history, and (d) no medication changes in the preceding six months. The study recruited 12 participants in an outpatient setting: six participants with normotensive diastolic BP (≤80 mmHg) at baseline and six participants with elevated diastolic BP (>80 mmHg) at baseline. The male-to-female ratio was 1:2. The participants aged between 52 and 73 years.

Product formulation

Vascanox HP® is a proprietary formulation that combines dietary nitrates in the form of beetroot extract with a source of hydrogen sulfide (black garlic extract), known reductants (vitamin C), various berry extracts (black currant extract, bilberry extract, raspberry extract, blue honeysuckle extract, and blueberry extract), and other vitamins and essential metals. A serving dose of two capsules of Vascanox® contains 242 mg of nitrates, which is equal to 109% of the acceptable daily intake (ADI).

Study protocol

The study participants were instructed to self-administer two capsules of Vascanox HP® each morning. They were evaluated in the clinic at baseline, at two weeks, and again at four weeks of study. The study lasted four weeks. During evaluation, the BP was measured in the branchial artery three times, five minutes apart. Salivary levels of NO were assessed using commercially available FDA-registered strips (NO Saliva Test Strips, MyFitStrip® LLC, Rockville, USA) prior to and two hours after dosing. NO test strips measure nitrite, which is used as an established surrogate marker for NO and nitrate [20]. Salivary NO was additionally assessed at six and 24 hours after dosing on the first day of the study only. They were asked to refrain from using mouthwash and products containing chlorhexidine or from taking additional supplements. Any medications were maintained with no changes for the duration of the study.

Statistical analysis

Descriptive statistics were performed to characterize the study sample. Analysis of variance (ANOVA) and paired t-tests were conducted to determine changes in study outcomes across time points. Subgroup analyses were performed among the subsamples with systolic hypertension and diastolic hypertension. Statistical significance was defined as p < 0.05. Statistical analyses were performed with SAS version 9.4.1 (SAS Institute Inc., Cary, NC). Additional analysis was performed using GraphPad Prism 9.0 (GraphPad Software, San Diego, CA).

## Results

Study population and design

The study population of 12 subjects consisted of 33% males (four subjects) and 77% females (eight subjects) between the age of 52-73 years. Systolic blood pressure (SBP) ranged between 127 and 141 (134 ± 4; mean ± SD) mmHg and was defined as elevated blood pressure in all subjects. Diastolic blood pressure (DBP) ranged between 62 and 94 mmHg for the 12 subjects where normotensive blood pressure was defined below 80 mmHg for DBP consisting of six subjects (50% of the study group) with a range of 62-74 (66 ± 5; mean ± SD) mmHg and six subjects (50% of the study group) were defined as elevated DBP with a range of 80-94 (84 ± 5) mmHg.

**Figure 1:**
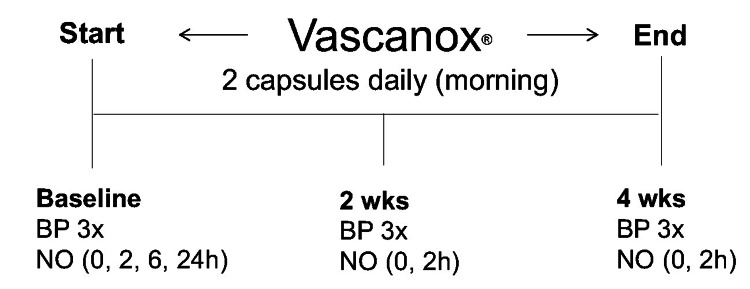
Study design Subjects were evaluated in the clinic at baseline, at two weeks, and again at four weeks of the study. After baseline assessment in the clinic, the study participants were instructed to self-administer two capsules of Vascanox® HP each morning thereafter. The study lasted four weeks. During the evaluation, the blood pressure (BP) was measured in the branchial artery three times (BP 3x), five minutes apart. Salivary levels of nitric oxide (NO) were assessed using NO Saliva Test Strips (MyFitStrip® LLC) prior to and two hours (h) thereafter. Salivary NO was additionally assessed at six and 24 hours after dosing on the first day of the study only.

The study design is summarized in Figure 1. The reported compliance rate was 100%. All participants adhered to the protocol and completed the study. Vascanox® HP was well-tolerated and no adverse effects were reported.

Salivary nitric oxide levels

**Figure 2:**
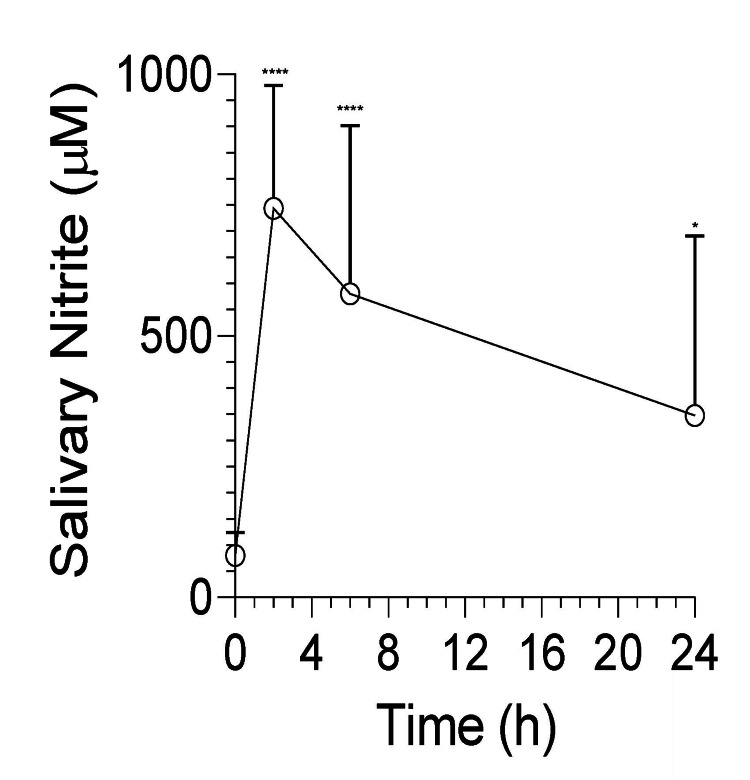
Salivary nitrite time course before and after Vascanox HP® Subjects were tested for salivary nitrite using prebiotic nitrate and nitrite saliva test strips by MyFitStrip® according to the methods. Upon testing at 0 minutes, subjects were given two capsules of Vascanox HP® according to the methods and subsequently tested at two, six, and 24 hours. **** p < 0.001; * p < 0.01.

Measurements of salivary nitrite (NO2-) and nitrate (NO3-) levels at the start of the clinical trial showed that Vascanox HP® administration led to a rapid increase in salivary NO2- (Figure 2) and NO3- (data not shown). Salivary NO2-, a surrogate of NO bioavailability, had an average value of 80 ± 44 uM (range: 20-110 uM; median:110 uM) at baseline to 743 ± 235 uM (range: 220-870 uM; median: 870 uM) at two hours post-dosing, as shown in Figure 2. Salivary NO2- levels decreased gradually to an average value of 580 ± 322 uM (range: 110-870 uM; median: 653 uM) at six hours and 348 ± 343 uM (range: 20-870 uM; median: 220 uM) at 24 hours. The measurements indicate that, without exception, participants experienced an increase in their salivary NO2- and NO3- levels with Vascanox® HP supplementation, with NO2- ranging between four-fold and 44-fold over baseline at two hours post-dosing. It was also observed that the coefficient of variation of salivary nitrite levels among participants at a specific time point increased from 30% at two hours to 60% at six hours and 100% at 24 hours post-supplementation, possibly due to genetic variations between individuals that influence the kinetics of nitrate clearance from circulation, nitrite metabolism through variation of the oral microbiome community responsible for the reduction of NO2- to NO in the oral cavity.

**Figure 3:**
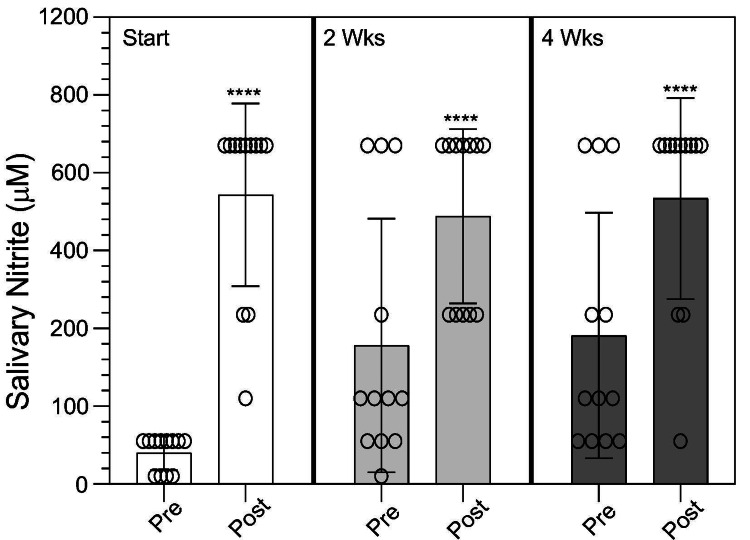
Salivary nitrite before and after two hours upon the start of the trial, and two and four weeks after daily oral administration of Vascanox HP® Subjects were tested for salivary nitrite using prebiotic nitrate and nitrite saliva test strips by MyFitStrip® according to the methods. Upon testing at 0 minutes, subjects were given two capsules of Vascanox according to the methods and subsequently tested at two hours post-administration on days 0, 14, and 28 with daily Vascanox administration. *** p < 0.001; ** p < 0.01.

Additional testing showed that daily Vascanox HP® supplementation produced similar increases in salivary NO2- concentration at two hours post-dosing at two and four weeks of study as it did on the first day of the trial (Figure 3). At two weeks, the pre-dosing salivary NO2- levels were 356 ± 325 uM (range: 20-870 uM; median: 220 uM) compared to 689 ± 224 uM (range: 435-870 uM; median: 870 uM) post-dosing. At four weeks, the pre-dosing salivary nitrite levels were 382 ± 315 uM (range: 110-870 uM; median: 110 uM) compared to 734 ± 258 uM (range: 110-870 uM; median: 870 uM) post-dosing. The measurements suggested that daily Vascanox HP® supplementation did not significantly alter nitrate/nitrite metabolism as average values and coefficient of variations observed at 24 hours post-dosing after the first Vascanox® HP administration were similar to those observed pre-dosing at two weeks and then at four weeks of the study.

BP measurements

**Figure 4:**
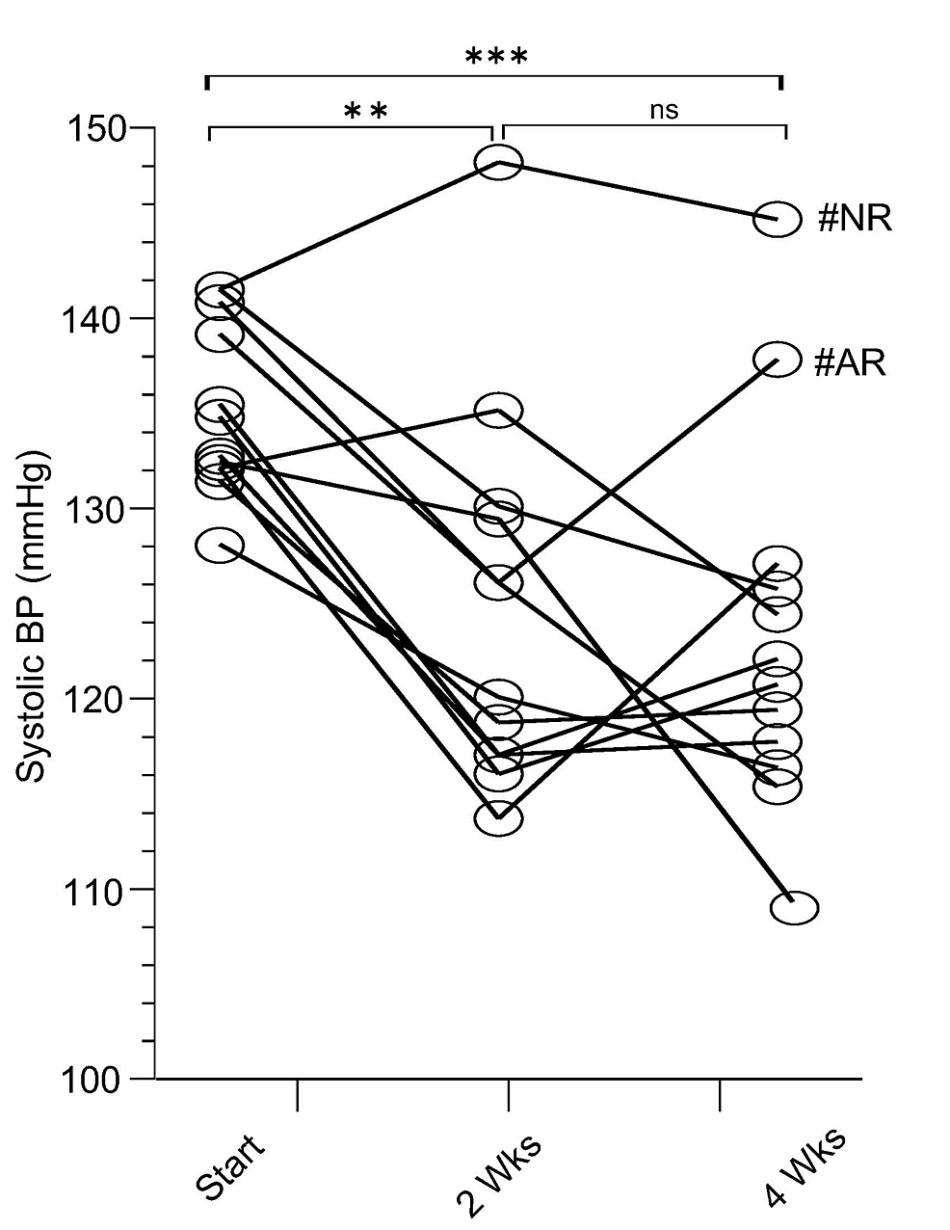
Systolic blood pressure before (start) and post daily administration of Vascanox HP® at days 14 and 28 Two individuals were classified as non-responders from the start (AR and NR) upon week four. All subjects were given two capsules of Vascanox daily, according to the methods, and systolic blood pressure was tested on days 14 and 28. *** p < 0.001; ** p < 0.01; ns: not statistically significant; BP: blood pressure.

Daily Vascanox® HP administrations decreased SBP values in 10/12 participants, as shown in Figure 4. One hypertensive individual (140/95 mmHg at baseline) did not experience a drop in SBP despite exhibiting significantly elevated salivary NO2- concentrations throughout the study. This is suggestive of a patient population that is naturally resistant to nitrate supplementation. Another mildly hypertensive participant (140/81 mmHg at baseline) experienced a reversal at four weeks after initially responding to Vascanox® HP with a decrease of 15 mmHg in SBP at two weeks of treatment (Figure 4).

In Figure 4, these two individuals are identified as #NR and #AR, respectively. Over the entire population, daily administration of Vascanox® HP led to a decrease of 10 mm Hg (p < 0.01) in SBP at two weeks of study and 11 mmHg (p < 0.001) at four weeks. Specifically, the mean SBP of the population dropped from 134 ± 4 (127-141) mmHg at baseline to 124 ± 10 (113-147) mmHg at two weeks and then to 123 ± 10 (115-144) mmHg at four weeks. The difference in SBP between two and four weeks was not statistically significant suggesting that Vascanox® HP is quick-acting in reducing SBP, which is maintained, at least as long as the supplementation persists.

**Figure 5:**
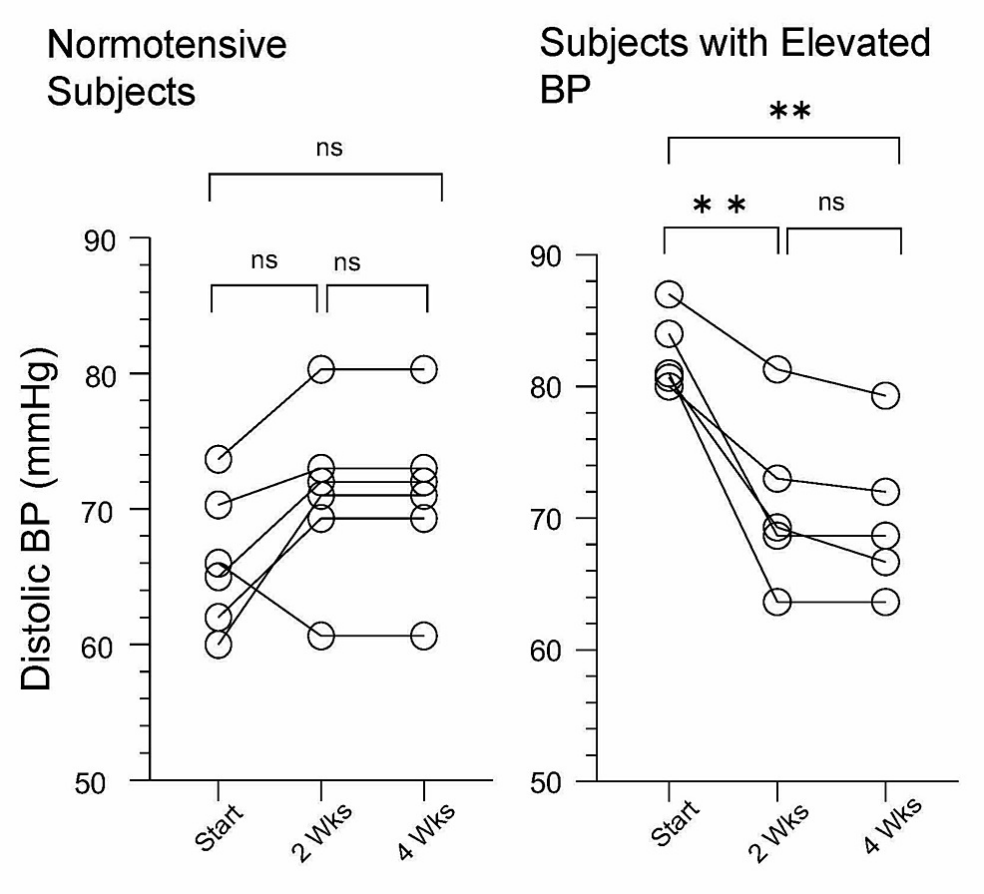
Diastolic blood pressure before (start) and post daily administration of Vascanox HP® at days 14 and 28 All subjects were given two capsules of Vascanox daily, according to the methods, and diastolic blood pressure was tested on days 14 and 28. There was no significant (ns) change among normotensive subjects whereas for those with elevated diastolic blood pressure, a statistically significant difference was found after two weeks of daily administration. *** p < 0.001; ** p < 0.01; BP: blood pressure.

BP measurements indicated that only participants with elevated DBP experienced a statistically significant decrease, whereas participants with DBP in the normotensive range did not. This is illustrated in Figure 5. Specifically, in study participants with DBP > 80 mmHg, a decrease of ~10 mmHg (p < 0.01) was recorded at two weeks and ~11 mmHg (p < 0.01) at four weeks of supplementation. Specifically, a mean DBP of 84.4 ± 5.4 (81-94) mmHg was noted at baseline, which then dropped to 74.5 ± 10.0 (69-91) mmHg at two weeks and 73.2 ± 8.4 (63-86) mmHg at four weeks.

The difference in SBP between two and four weeks was not statistically significant for the group; this suggests again that Vascanox® HP is quick-acting. The hypertensive patient, whose SBP did not respond to Vascanox® HP supplementation, experienced a drop in DBP from 94 mmHg at baseline to 91 mmHg at two weeks and 86 mmHg at four weeks. For normotensive participants, the mean DBP was 66.2 ± 5.4 (62-74) mmHg at baseline, 71.1 ± 6.4 (60-80) mmHg at two weeks, and 68.3 ± 6.1 (57-74) mmHg at four weeks. For normotensive subjects, the differences were statistically significant.

## Discussion

This open-label clinical trial evaluated the safety and efficacy of Vascanox® HP, a proprietary formulation designed to restore the bioavailability of gaseous transmitters NO and hydrogen disulfide in individuals with elevated BP or mild hypertension. The clinical trial raised no safety issues and demonstrated that short-term supplementation with Vascanox® HP leads to statistically significant decreases of 10-11 mmHg in both SBP and DBP. Interestingly, participants who exhibited elevated SBP but not DBP experienced a decrease only in their SBP. This reinforces the safety of Vascanox® HP as a dietary supplement and may be suggestive that there is crosstalk between the canonical (eNOS) and non-canonical (dietary nitrate) pathways of NO synthesis with the latter becoming important when the former is dysfunctional.

Since the identification of dietary nitrates as a source of NO, numerous clinical trials have been performed, which examined the effect of nitrate supplementation on SBP and DBP. In early clinical trials, nitrates were administered as inorganic salts or beetroot juice to healthy volunteers, whereas later trials explored combinations of concentrated beetroot powder, sodium nitrite, and various phytochemicals given to patients diagnosed with elevated BP [21-25]. In 2013, Siervo et al. presented a meta-analysis of 16 early, randomized, placebo-controlled clinical trials. The trials were performed between 2006 and 2012 and involved a total of 254 participants, who consumed between 157 and 1488 mg of nitrate [21]. The investigators of the meta-analysis concluded that inorganic nitrate and beetroot juice consumption were associated with a statistically significant change of ~4.4 mmHg in SBP. The effect on DBP was much smaller (~1.1 mmHg) and did not reach statistical significance [21].

More recent clinical trials on dietary supplements formulated with nitrate or nitrite decreased SBP and DBP in mostly prehypertensive subjects. A complex formulation by Berkeley Life®, consisting of encapsulated beetroot extract, thiamine nitrate, potassium nitrate, and ascorbic acid, showed a decrease of 6.3 mmHg in SBP and 4.7 mmHg in DBP when given daily to individuals with baseline BP of >120/80 mmHg for 12 weeks [25]. The source of NO from this formulation is primarily dependent on NO3- derived from potassium nitrate and to a lesser extent from thiamine mononitrate and the beet extract. This formulation is approximately 5 mM nitrate, in the absence of any nitrite or NO2-, and the durability of a single dose over the course of eight to 24 hours is unclear.

Another supplement, Neo40®, was evaluated on the acute effects of a formulation containing sodium nitrite with a reported decrease of 4 mmHg in SBP and 5 mmHg in DBP, respectively, within 20 minutes [22]. Neo40® is a disintegrating lozenge that contains sodium nitrite admixed with nitrite reductase in the form of hawthorn berry extract, L-citrulline, and beetroot powder consisting of low-mM nitrate [22]. The rapid reduction of sodium nitrite appears to be the primary source of NO generated from Neo40® and the durability beyond six to eight hours with a single dose was not evaluated.

A significant decrease in SBP and DBP was reported by Baik et al., who used a garlic extract fermented to contain nitrite [26]. In that study, when the investigators compared the release of NO in simulated gastric fluid from a single tablet of fermented garlic extract (FGE) containing 7 mg nitrite with the corresponding release of NO from a single lozenge of Neo40®, they found that the FGE tablet released 89-fold more NO than the Neo40® lozenge despite the lower concentration of nitrite in the FGE tablet [26]. They also showed that not only the peak levels of NO were higher with the FGE tablet, but also the NO release lasted almost 10 hours compared to 30-60 minutes with the Neo40® lozenge. It was postulated that different compositions of polyphenols and/or reductants present in the supplements were largely responsible for the differences in NO production between the FGE and Neo40® [26]. Since nitrite (unlike nitrate) itself is relatively short-lived, the fermented garlic had a significant effect on the generation and durability of NO as suggested by Baik et al. [26].

To our knowledge, Vascanox® HP is the first commercial dietary supplement that, inspired by recent advances in the field of NO biology, combines dietary sources of NO and H2S. Unlike Neo40®, whose nitrite contents per daily serving exceed the established ADI of 0.07 mg/kg/day by 10-fold, Vascanox® HP contains no nitrite and relies on commensal oral bacteria for the reduction of NO3- to NO2-, a necessary and required step in the well-established enterosalivary loop [9,10]. Its dietary NO3- content of ~240 mg/per daily serving is close to the ADI limit for NO3- and far below the typical dietary nitrate content found, for example, in beetroot juice, which can contain up to 11.4 g/L of nitrates [27].

**Figure 6:**
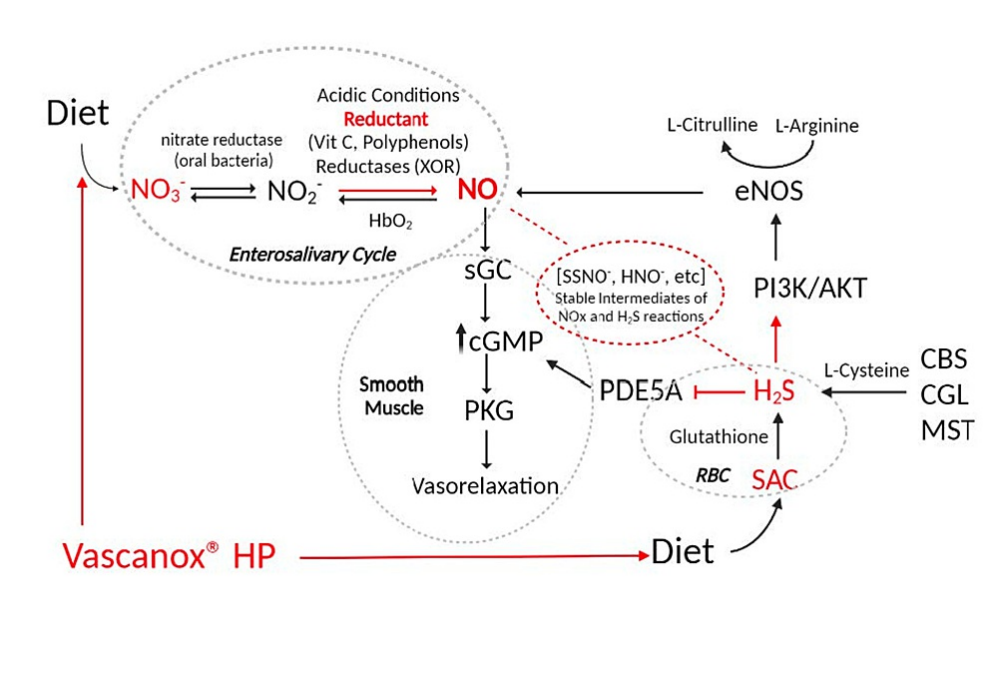
Pathways controlling nitric oxide synthesis and vasorelaxation. Hypothetical points of Vascanox® HP intervention are in red. NO: nitric oxide; Vit C: vitamin C; XOR: xanthine oxidoreductase; HbO2: oxyhemoglobin; eNOS: endothelial nitric oxide synthase; SSNO-: nitrosopersulfide; HNO-: nitroxyl; NOx: nitrogen oxides to include nitrite, nitrate, and reactive nitrogen oxide intermediates; H2S: hydrogen sulfide; sGC: soluble guanylate cyclase; cGMP: second messenger cyclic; PKG: protein kinase G; PDE5A: phosphodiesterase type 5; PI3K/AKT: phosphoinositide 3-kinase/protein kinase B; CBS: cystathionine β synthase; CGL: cystathionine γ-lyase; MST: 3-mercaptopyruvate sulfurtransferase; SAC: S-allylcysteine; RBC: red blood cell. This figure was created by the authors. Under open-access Creative Commons, this figure may be reused without permission provided that this article is cited.

Despite its reduced dietary nitrate content and absence of added nitrite salts, Vascanox® HP supplementation demonstrated efficacy in reducing BP. We attributed that effect to the ability of its complex formulation to generate and sustain elevated levels of NO. As suggested in Figure 6, the extended NO bioavailability might be credited to the presence of black garlic, which, as a dietary source of hydrogen sulfide, is expected to amplify eNOS activation and NO synthesis through both the canonical and non-canonical pathways, to maintain elevated levels of cGMP, and to possibly combine with nitrites to form stable intermediates that serve as NO stores, i.e., found in muscle [28]. Furthermore, vitamin C and polyphenols present in the Vascanox® HP formulation might also act as reductants assisting in the conversion of nitrite to NO in the enterosalivary cycle, thus preventing NO oxidation and renal excretion as a nitrate. Rocha et al. have shown that, in the presence of nitrite, dietary polyphenols elevate NO levels in the stomach with the magnitude of the increase being dependent on the type and concentration of polyphenols [29]. Vascanox® HP contains an advanced blend of berry extracts in addition to beetroot powder, black garlic, vitamin C, and metals known to influence NO biosynthesis. Because of the particular combination of ingredients, Vascanox® HP is expected to affect both canonical and non-canonical pathways of NO production and to augment NO reservoir, i.e., muscle, in the body, leading to an increased and prolonged NO bioavailability. The collective effect of Vascanox® HP is an impressive reduction in SBP and DBP in mildly hypertensive individuals.

Limitations

The study was limited by the small number of participants and the open-label design. A placebo effect influencing the degree of BP reduction in the study subjects cannot be excluded. Furthermore, the clinical trial did not include measurements of the circulating concentrations of NO and sulfides, which would have been helpful in determining peak NO levels in the blood, the duration of its bioavailability, and the possible effect of the inclusion of black garlic as an ingredient in Vascanox® HP. The study would also have benefited from experiments examining NO release in gastric simulants. Finally, the study tested sub-chronic supplementation and did not report the development of resistance in the participants with the possible exception of a single recruit. It is apparent that a larger number of participants and longer supplementation are needed to exclude the possibility that a certain percentage of subjects will develop resistance to Vascanox® HP in future studies.

## Conclusions

This open-label clinical trial evaluated the safety and efficacy of Vascanox® HP, a proprietary formulation designed to restore the bioavailability of gaseous transmitters NO and hydrogen disulfide in individuals with elevated BP or mild hypertension. The clinical trial raised no safety issues and demonstrated that short-term supplementation with Vascanox® HP leads to statistically significant decreases of 10-11 mmHg in both SBP and DBP. Interestingly, participants who exhibited elevated SBP but not DBP experienced a decrease only in their SBP. This reinforces the safety of Vascanox® HP as a dietary supplement and may be suggestive that there is crosstalk between the canonical (eNOS) and non-canonical (dietary nitrate) pathways of NO synthesis with the latter becoming important when the former is dysfunctional.
